# Age and the digital divide in the use of health technologies: a scoping review

**DOI:** 10.3389/fdgth.2026.1786370

**Published:** 2026-08-13

**Authors:** Lowryanne Vick, Christina M. Armstrong, Cathy Cruise

**Affiliations:** Connected Care, Digital Health Office, U.S. Department of Veterans Affairs, Washington, DC, United States

**Keywords:** digital divide—ageing—peer training—aged care—digital inclusion, digital health (DH), older adult, scoping review, telehealth (TH)

## Abstract

**Introduction:**

The present study undertakes a scoping review of research on the intersection of age, health technologies, and the digital divide, with a specific focus on identifying facilitators and barriers to adoption, and strategies for inclusive implementation.

**Methods:**

A scoping review of literature from ten databases was used to identify papers published 2010–2024, from which we selected 45 publications that focused on older adults, technology, and health care. Technologies included synchronous video, asynchronous telehealth, wearable sensors, mobile health devices, and other health technologies across four areas of impact: individual, clinic, hospital, and system or organizational levels. Qualitative content, framework analyses, and subject matter expert discussion were used to analyze data and identify best practices in implementation.

**Results:**

Of the 4,368 potential literature references, 264 were duplicates, and our review of the title and abstracts of 4,104 (93.9%) found that 3,692 (90.0%) were irrelevant. A full-text review of 412 studies revealed that 83 (20.1%) studies met all the criteria related to technology and health care, and 38 (45.7%) were removed (17 search terms were mentioned yet not a focus of the research, 12 included methods not sufficiently geared to assess outcomes, 5 were non-peer reviewed literature and 4 had the wrong publication type (e.g., commentary, editorial)). This resulted in 45 articles from seven countries and one global/multinational study ranging from 2017 to 2024. All studies addressed the individual level (*n* = 45, 100.0%). Focus areas of articles were individual (*n* = 45, 100%), unit/team level (*n* = 8, 17.8%), site/facility level (*n* = 5, 11.1%), and system level (*n* = 8, 17.8%), with studies increasing focus on multiple areas over time.

**Conclusions:**

This scoping review highlights a growing body of literature exploring the intersection of aging, healthcare, and digital technology, but also reveals gaps in addressing multi-level implementation challenges.

## 1 Introduction

The aging population in the United States is growing at an unprecedented rate, with adults aged 65 and older projected to make up nearly 25% of the population by 2060 (1). This demographic shift is accompanied by an increasing burden of chronic conditions which significantly contributes to morbidity, disability, and rising healthcare costs (2). Older adults are also at higher risk for geriatric syndromes like frailty, falls, and cognitive decline, further complicating their healthcare needs (3).

This issue is particularly relevant for older Veterans, who face unique challenges due to both age-related conditions and service-connected disabilities. Nearly half of the 18 million U.S. Veterans are 65 or older, and by 2028, one-third of Veterans will be 75 or older (4). Older Veterans often experience higher rates of multimorbidity, and functional impairments compared to their non-Veteran counterparts, making them more susceptible to adverse health outcomes (5–7). The increasing prevalence of service-connected disabilities further underscores the importance of healthcare solutions tailored to their specific needs. While older Veterans represent a particularly compelling example of the complex healthcare needs that can accompany aging, these challenges are not unique to this population. The management of chronic conditions, functional decline, and access to care reflect broader trends affecting older adults across the United States and underscores the need for scalable, inclusive healthcare solution.

Although aging may be associated with a gradual decline in health, each person ages differently. Medical advances are increasing life expectancies and positively impacting health. Psychological, lifestyle, and sociocultural factors shape diverse aging trajectories, underscoring the need to avoid stereotypes of older adults as uniformly ill or disabled. Many older adults actively manage their health, despite chronic conditions. Experts recommend strategies to support healthy aging, highlighting the need for creative approaches utilizing technology to improve access and outcomes.

Throughout this manuscript, we use the term telehealth as an umbrella term encompassing the delivery of health-related services and information via digital communication technologies, which the literature also variously refers to as telemedicine, digital health, eHealth, virtual care, and health technology; unless quoting directly from cited sources, we adopt telehealth as our consistent terminology. Telehealth represents an innovative approach to healthcare delivery that improves access, reduces caregiver burden, and decreases hospitalizations and emergency department visits (8, 9). It alleviates travel-related challenges linked to mobility, cognitive, or transportation limitations, enables more timely and frequent provider interactions, enhances caregiver engagement, supports interdisciplinary collaboration, and promotes independent living and aging in place (8–11).

Older adults are increasingly embracing these technologies and challenging common stereotypes. In 2021, 75% of older adults used the internet and 44% owned a tablet, with nearly three-quarters accessing the internet daily (12). Among Veterans, high rates of device ownership and home internet access have similarly been noted (13). Older adults value telehealth for its convenience, cost savings, and lower infection risk, and many actively seek to improve their digital literacy to better access healthcare (14, 15). The dramatic rise in telehealth use among Medicare beneficiaries, from approximately 13,000 weekly visits before the COVID-19 pandemic to nearly 1.7 million by late April 2020, further underscores older adults' growing comfort with and acceptance of telehealth (8, 9). As digital familiarity rises, telehealth becomes a more viable option, especially for those with barriers to in-person care.

Telehealth facilitates meeting the needs of older adults by aligning closely with the four evidence-based elements of high-quality care embodied in the 4Ms Framework (16) and by empowering individuals to actively manage their health, maintain functional independence, strengthen social connections, and support aging in place. Despite its promise, widespread and equitable implementation remains hindered by the digital divide, disparities in access, digital literacy, and engagement, compounded by unique challenges older adults face related to usability, accessibility, technology readiness, and bias by healthcare staff and systems. Bridging this divide is essential to ensure older adults receive timely, effective, and patient-centered care.

Although research on aging and digital health is expanding, the scope, characteristics, and gaps in this literature remain unclear. This scoping review systematically mapped existing studies to identify key concepts, barriers, facilitators, and strategies for inclusive implementation. It examined study designs, populations, settings, technologies, functions, and conceptual frameworks, with attention to how older adults are defined and represented. The review also explores evaluation tools, levels of analysis, and how these elements have evolved. Findings aim to inform evidence-based innovation and highlight critical gaps at the intersection of aging, technology, and the digital divide.

## 2 Methods

### 2.1 Study design and search strategy

The original six-stage process (17) and updated modifications (18) for scoping reviews were used. The literature key word search was conducted for articles published through December 2024 using PubMed/Medline, American Psychological Association (APA) PsycNET, Cochrane, Embase, PsycINFO, Web of Science and Scopus, Science Citation Index, Social Sciences Citation Index, Telemedicine Information Exchange database, Centre for Reviews and Dissemination, and The Cochrane Library Controlled Trial Registry database.

Where available, database-specific controlled variables (MeSH terms in PubMed/MEDLINE; Emtree in Embase; Thesauraus terms in PsychINOFO) were used in combination with free-text keywords to maximize sensitivity. The four core concept areas and their associated terms were:

Concept 1 - Older Adults: elderly, aging, geriatric, seniors, “older adults”
Concept 2 - Health Technology (General/Asynchronous): mobile, android, app, asynchronous, cellular, device, ehealth, “e-health,” ebehavioral, “e-behavioral,” “e-mental health,” mhealth, “m-health,” phone, smartphone, tablet, text, eConsult, “e-consult,” email, “e-mail,” monitoring, sensor, “social media,” “store-and-forward,” wearable
Concept 3 - Synchronous Technology: telehealth, “telebehavioral health,” “telemental health,” video, “web-based,” Internet
Concept 4 - Digital Divide: “digital equity,” “digital literacy,” “age discrimination,” “digital divide”.

### 2.2 Study selection

**Figure 1:**
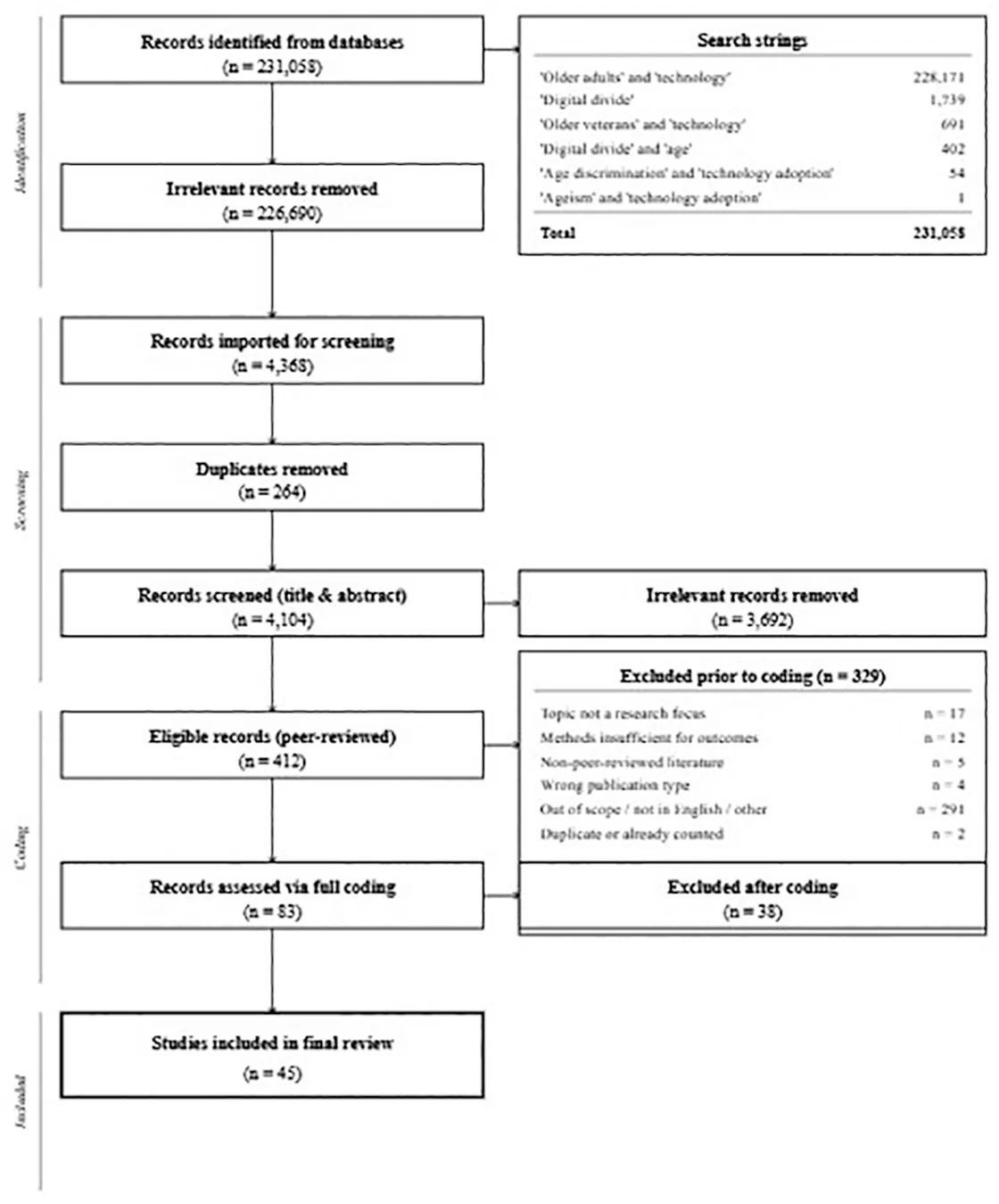
Search flow chart: diagram of papers reviewed, excluded and selected.

Three authors (LV, CA, CC) conducted the database searches. Title and abstract screening were performed independently by two authors (LV, CA), with a third author (CC) consulted to resolve discrepancies and reach consensus. Full-text articles were then reviewed independently by two authors (LV, CA) to determine final inclusion based on the eligibility criteria. Any discrepancies arising during full-text review were resolved through discussion among all three authors (LV, CA, CC) until consensus was reached. Reviewers also met at the beginning, midpoint, and final stages of the review to discuss challenges and uncertainties and to refine the search strategy as needed. The full search and selection process is illustrated in Figure 1.

Inclusion criteria were based on search terms used in combination across four concept areas: 1 and 2; 1 and 3; or 1, 2, and 3. Exclusion criteria included the following: terms used out of context (e.g., abstract use of competency not referring to a measurable behavior or skill); data unrelated to age or technology; content not applicable to clinical care; wrong report type (e.g., abstract only, editorial, column); models, principles, or concepts with no educational outcomes; non-English language publications; and other reasons deemed outside the scope of the review.

### 2.3 Data extraction and reporting

A data charting form was developed in Excel (Version 2402; 19) and calibrated within the review team prior to extraction. The following variables were charted for each included study: author(s), publication year, country, study design, population characteristics, technology type, level of analysis (individual, team/unit, facility, or system), barriers to technology adoption, facilitators of technology adoption, and reported outcomes. Data charting was performed independently by two authors (LV, CA), and any discrepancies were resolved through discussion with a third author (CC) until consensus was reached. The reviewers compared and consolidated information using a qualitative content analysis approach (20).

## 3 Results

The database search yielded 228,171 records from searches combining “older adults” and “technology,” 1,739 from “digital divide,” 691 from “older veterans” and “technology,” 402 from “digital divide” and “age,” 54 from “age discrimination” and “technology adoption,” and 1 from “ageism” and “technology adoption.” Following an initial import review, 226,690 irrelevant records were removed, yielding 4,368 records imported for screening. After 264 duplicates were removed, 4,104 records were screened by title and abstract, of which 3,692 were excluded as irrelevant, leaving 412 eligible records comprising peer-reviewed, original research including conference proceedings.

Of the 412 eligible records, 329 were excluded prior to coding for the following reasons: search terms were mentioned but the topic was not a focus of the research (*n* = 17); methods were not sufficiently geared to assess outcomes (*n* = 12); non-peer-reviewed literature such as books, chapters, or grey literature (*n* = 5); wrong publication type such as abstracts, columns, commentaries, or editorials (*n* = 4); and out of scope, not in the English language, or other reasons (*n* = 291). The remaining 83 articles proceeded to full coding. Following coding, an additional 38 articles were excluded, resulting in a final sample of 45 studies included in the review. Additional information on the search and selection process is available in Figure 1.

Of records that were coded, 45 met all criteria and are reported in Table 1.

**Table 1:** Findings in aging and virtual care in healthcare.

#	Author, Year	*N* (participants)	Length of study	Healthcare staff population	Special patient populations description (if applicable)	Country	Design	Technology (hardware, software?)	Individual level	Unit/team level	Site/facility level	System level
1	Alfaro et al. (21)	17	2.5 years	Mental health clinicians and trainees (i.e., psychology practicum students, interns, post-doctoral fellows, clinical psychologists)	Veterans, mobility and mental health	United States	Quality improvement study/Feasibility Pilot Study	Mobile/web apps, smartphone/tablet, synchronous and asynchronous telehealth	X			
2	Bahar-Fuchs et al. (22)	84	2 years	n/a	Diabetes at risk for dementia	Global	Randomized controlled trial	Computer, mobile/web apps, smartphone/tablet, synchronous and asynchronous telehealth	X			
3	Bahar-Fuchs et al., (69)	44	12 weeks	n/a	Mild cognitive impairment (MCI), mood-related neuropsychiatric symptoms (MrNPS), or both	Australia	Randomized controlled trial	Computer, mobile/web apps, smartphone/tablet, synchronous and asynchronous telehealth	X			
4	Bhatia et al. (23)	208	2 years	Primary care providers	n/a	United States	Mixed methods study	Synchronous telehealth	X			
5	Boudreau et al. (24)	30	4 months	Interdisciplinary geriatric specialty team and caregivers	Rural Veterans	United States	Cross-sectional study	Synchronous telehealth	X	X	X	X
6	Chang et al.(25)	5,437	6 months		n/a	United States	Cross-sectional study	Synchronous telehealth	X			
7	Choxi et al.(26)	13	1 month	Healthcare staff (general)	n/a	United States	Qualitative descriptive study	Synchronous telehealth	X	X	X	X
8	Chu et al. (27)	1,025	3 months	General internal medicine primary care physicians and staff	n/a	United States	Quality improvement study/Feasibility Pilot Study	Synchronous telehealth	X			
9	Dang et al. (28)	602	1 year	n/a	Veterans with high medical needs and at high risk	United States	Cross-sectional study	Synchronous and asynchronous telehealth	X			
10	Dawson et al. (30)	74	12 weeks	Two staff members (serving as support staff to assist with resolving technical difficulties or other urgent matters during each virtual class) including one certified Tai Chi instructor	Veterans experiencing loneliness and mobility issues	United States	Quality improvement study/Feasibility Pilot Study	Synchronous telehealth	X			
11	Dukhanin et al. (31)	73	2 years	Clinicians and clinic staff, medical informatics teams, marketing and communications staff, administrators, funders, and thought leaders.	n/a	United States	Qualitative descriptive study	Computer, mobile/web apps, smartphone/tablet, synchronous and asynchronous telehealth	X	X	X	X
12	Freytag et al. (32)	14	6 months	Geriatrics outpatient healthcare staff	Veterans with dementia	United States	Quality improvement study/Feasibility Pilot Study	Mobile/web apps, smartphone/tablet, synchronous and asynchronous telehealth	X			
13	Frydman et al. (33)	6,813	6 years	n/a	Serious illness	United States	Cross-sectional study	Computer, mobile/web apps, smartphone/tablet, synchronous and asynchronous telehealth	X			
14	Gately et al. (34)	25	3 months	n/a (caregivers)	Dementia	United States	Qualitative descriptive study	Synchronous telehealth	X			
15	Goldberg et al. (35)	48	3 months	Physicians	n/a	United States	Qualitative descriptive study	Synchronous telehealth	X			X
16	Gould et al. (13)	77	2.5 years	n/a	Veterans	United States	Mixed methods study	Computer, mobile/web apps, smartphone/tablet, synchronous and asynchronous telehealth	X			
17	Haimi and Sergienko (36)	618,850	2.5 years	Non-specific	n/a	Isreal	Retrospective cohort study	Synchronous and asynchronous telehealth	X	X	X	X
18	Harris et al. (37)	14	24 weeks	Physical therapists	Veterans with mobility issues	United States	Quality improvement study/Feasibility Pilot Study	Synchronous telehealth	X			
19	Hastings et al. (38)	40	1.5 years	Nurses	Veterans with complex medical conditions and cognitive impairment.	United States	Randomized controlled trial	Synchronous telehealth	X			
20	Hawley et al. (39)	20	1.5 years	Pharmacists	Veterans with at least two chronic medical conditions, but no dementia diagnosis	United States	Qualitative descriptive study	Synchronous telehealth	X			X
21	Huang et al. (40)	180	3 months	Personal trainers and yoga instructors	Urinary incontinence	United States	Randomized controlled trial	Synchronous telehealth	X			
22	Iyer et al. (41)	30	1.5 years	n/a (caregivers)	Veterans with dementia	United States	Qualitative descriptive study	Synchronous telehealth	X			
23	Iyer et al. (42)	386	4.5 years	Primary care providers (physicians and nurse practitioners), social workers, nurses, neuropsychologists, and geriatricians	Veterans with dementia	United States	Quality improvement study/Feasibility Pilot Study	Synchronous and asynchronous telehealth	X			X
24	Jeste et al. (43)	20	18 weeks	n/a	n/a	United States	Randomized controlled trial	Synchronous telehealth	X			
25	Koch et al. (44)	254	2 years	Mental health clinician (attending physician, resident physician, nurse practitioner, or physician assistant	Mental health issues	United States	Retrospective cohort study	Synchronous telehealth	X			
26	Lee et al. (45)	505	4 weeks	n/a	n/a	South Korea	Cross-sectional study	Mobile/web apps, smartphone/tablet, synchronous and asynchronous telehealth	X			
27	Mannheim et al. (46)	97	n/a	Physiotherapists	n/a	Netherlands	Mixed methods study	Health technology (general)	X			
28	Mao et al. (47)	2,607	10 years	General practitioner, pharmacist, hospital doctor, or other healthcare professional	n/a	Ireland	Cross-sectional study	Synchronous telehealth	X			
29	Martinson et al. (48)	141	1.5 years	Primary care physicians and staff	Veterans with chronic pain	United States	Mixed methods study	Synchronous telehealth	X			
30	McQuown et al. (49)	247	6 months	Intermediate care technicians, geriatricians, emergency room physicians	Veterans	United States	Quality improvement study/Feasibility Pilot Study	Synchronous telehealth	X	X		
31	Mitzner et al. (50)	14	n/a	Exercise instructors	Arthritis and mobility issues	United States	Mixed methods study	Synchronous telehealth	X	X		
32	Nearing et al. (51)	50	3 months	Healthcare staff (general)	rural Veterans	United States	Quality improvement study/Feasibility Pilot Study	Synchronous telehealth	X	X	X	X
33	Odebunmi et al. (52)	1,045	n/a	Healthcare providers and pharmacists	n/a	United States	Cross-sectional study	Synchronous and asynchronous telehealth	X			
34	Orlofsky and Wozniak (53)	12	6 months	Nurses and other geriatric healthcare professionals	n/a	United States	Qualitative descriptive study	Voice-activated personal assistants	X			
35	Padala et al. (54)	118	4 weeks	Geriatrician (geriatrics and memory clinics)	n/a	United States	Cross-sectional study	Health technology (general)	X			
36	Park et al. (55)	14	6 weeks	n/a	dementia	United States	Mixed methods study	Computer, mobile/web apps, smartphone/tablet, synchronous and asynchronous telehealth	X			
37	Qin (56)	1,769	4 months	n/a	n/a	United States	Retrospective cohort study	Synchronous telehealth	X			
38	Rauzi et al. (57)	50	3 weeks	Physical therapists	Veterans with dementia	United States	Mixed methods study	Synchronous and asynchronous telehealth	X			
39	Repetti et al. (58)	14	2 months	n/a	n/a	Switzerland	Mixed methods study	Health technology (general)	X			
40	Schubert et al. (59)	4,996	10 years	Geriatricians, mental health psychologists, pharmacists, nurse practitioners, social workers	Veterans	United States	Retrospective cohort study	Synchronous telehealth	X			
41	Schwartz et al. (60)	1,895	2 years	Physicians, nurse practitioner, pharmacist, social worker interprofessional trainees	Veterans	United States	Qualitative descriptive study	Synchronous telehealth	X			
42	Stypulkowski et al. (61)	24	6 weeks	Primary care providers, licensed psychologists, and caregivers	Mild cognitive impairment	United States	Quality improvement study/Feasibility Pilot Study	Mobile/web apps, smartphone/tablet, synchronous and asynchronous telehealth	X	X		
43	Taylor et al. (62)	58	2 months	Occupational therapy practitioners	n/a	United States	Mixed methods study	Computer, mobile/web apps, smartphone/tablet, synchronous and asynchronous telehealth	X			
44	Thompson et al. (63)	13	8 weeks	Certified trainers who specialize in strength training for older adults	n/a	United States	Quality improvement study/Feasibility Pilot Study	Mobile/web apps, smartphone/tablet, synchronous and asynchronous telehealth	X			
45	Wardlow et al. (8)	7,246	10 days	Independent practitioners (i.e., MD, NP, or PA); nurses; mental or behavioral health providers; occupational or physical therapist	n/a	United States	Qualitative descriptive study	Synchronous and asynchronous telehealth	X			
Total #									45	8	5	8
Total %									100.0	17.8	11.1	17.8

A total of 45 articles were included in this review. Most studies (*n* = 38, 84.4%) were conducted in the United States. The remaining articles represented Australia, Ireland, Israel, the Netherlands, South Korea, Switzerland, and global/multinational efforts, each contributing one study (2.2%). The predominance of U.S. based studies may limit the transferability of findings to other countries. Differences in digital infrastructure, healthcare delivery models, policy environments, and population demographics may influence older adults' experiences with digital health technologies and contribute to variation in the barriers and facilitators identified across settings. Studies were published from 2017 to 2024, with an increase in the number of published studies over time indicating a growing interest in this area of research. Most studies were published in recent years, with 91.1% (*n* = 41) published between 2021 and 2024. Only 8.9% (*n* = 4) were published between 2017 and 2020. The years 2022 and 2024 had the highest number of publications (*n* = 16 each, 35.6%), followed by 2023 (*n* = 7, 15.6%).

Study length was reported in 42 of the 45 studies (93.3%). The mean study duration was 75.8 weeks (approximately 1.46 years), with a median of 25 weeks (0.48 years) and a range from 1.5 weeks to 10 years. The mode study duration was 13 weeks (0.25 years). All studies reported their design. The most frequent designs were quality improvement or pilot feasibility studies (*n* = 10, 22.2%), mixed methods (*n* = 9, 20.0%), qualitative descriptive (*n* = 9, 20.0%), and randomized controlled trials (*n* = 9, 20.0%). Cross-sectional designs were also present (*n* = 8, 17.8%).

### 3.1 Patient populations

Nearly all studies (*n* = 43, 95.6%) focused specifically on older adults, and a smaller number (*n* = 2, 4.4%) included both adults and older adults. Of the 36 studies that provided additional population details, 50% (*n* = 18) included veterans. Other commonly represented older adult populations included those with dementia (16.7%), those living in rural areas (5.6%), and individuals with mobility issues, mental health challenges (e.g., loneliness, depression, anxiety), or other medical conditions (e.g., diabetes, chronic pain, urinary incontinence, arthritis). Definitions of “older adult” varied across studies, with 60% (*n* = 27) defining older adults as those aged 65 years and older. Other thresholds included 60+ (20.0%, *n* = 9), 50+ (8.9%, *n* = 4), 55+ (6.7%, *n* = 3), and 70+ (4.4%, *n* = 2).

### 3.2 Use of frameworks, assessments, technology, and interventions

One-third of studies (*n* = 15, 33.3%) used at least one conceptual or theoretical framework. Among these, the mean number of frameworks per study was 1.6 (range = 1–4). A total of 25 frameworks were identified. The most common was the 4Ms of Age-Friendly Health Systems (*n* = 4, 16%), followed by the Theoretical Domains Framework (*n* = 2, 8%).

Sixty-seven percent of studies (*n* = 30) used at least one standardized assessment. Across all studies, 164 total assessments were used, comprising 136 distinct instruments. The average number of assessments per study was 5.3 (median = 3.5, mode = 1, range = 1–23). Commonly repeated tools included the PHQ-9 (*n* = 5), MoCA (*n* = 4), and various cognitive and functional assessments. Areas assessed included cognitive ability (30.9%), behavioral health symptoms (19.1%), and physical functioning (18.4%).

Of the technologies that were described in studies, synchronous telehealth was the most frequently reported technology type (*n* = 23, 51.1%). Other configurations included combinations of mobile/web apps, smartphones/tablets, asynchronous telehealth (*n* = 17, 37.8%), general health technologies (*n* = 3, 6.7%), and voice-activated personal assistants (*n* = 1, 2.2%).

Just over half of the studies (*n* = 25, 55.6%) described a specific intervention. Of these, healthcare delivery interventions were the most common (*n* = 8, 32.0%), followed by exercise programs (*n* = 6, 24.0%) and educational or consultation-based interventions (*n* = 4, 16.0%).

#### 3.2.1 Areas of focus

All studies addressed the individual level (*n* = 45, 100.0%). Additional focus areas included the unit/team level (*n* = 8, 17.8%), site/facility level (*n* = 5, 11.1%), and system level (*n* = 8, 17.8%). Studies from 2017 to 2021 only addressed individual level, but starting in 2022 studies began to focus on unit/team, site/facility, and system levels. The most diverse range of focus was observed in 2024, with 31.3% of studies addressing system-level issues and 18.8% focusing on site/facility level.

### 3.3 Findings from studies

Telehealth offers numerous advantages that enhance the quality, accessibility, and efficiency of healthcare for older adults, including support of proactive and longitudinal care by facilitating monitoring between in-person visits and improving management of acute conditions and transitions of care (8, 9). Through the use of telehealth, older adults have been shown to benefit from increased treatment adherence and access to mental and behavioral health services, while also being able to receive care across regions, including while aging in place (8, 9). Table 2 summarizes key findings from recent literature that highlight the multifaceted advantages of telehealth in the older adult population.

**Table 2:** Benefits of telehealth for older adults.

Category	Benefits	Citations
Access to care	Increased access to care; specialty services across regions/states; reduced travel burden; easier/faster access to providers; aging in place.	(8, 9, 35)
Clinical effectiveness	Improved acute illness management; improved transitions of care; fewer hospitalizations; reduced ED use; improved treatment adherence.	(8, 9)
Efficiency	Decreased time for specialty visits; fewer cancellations/no-shows; improved physician workflow; ability to offer repeat visits.	(8, 9, 35)
Cost savings	Reduced patient costs; reduced health system costs.	(8, 9)
Patient experience	Increased patient satisfaction; reduced stress for caregivers; reduced long wait times.	(8, 9, 35)
Safety and public health	Reduced exposure to infectious diseases; reduced spread of communicable illnesses.	(8, 9, 35)
Care coordination and communication	Enhanced provider–patient–family communication; caregiver involvement; support for longitudinal monitoring and proactive care.	(8, 9, 35)
Health equity and outreach	Facilitated outreach and education; reduced deferred care; increased timely care delivery.	(35)

Timely interventions through telehealth reduce the need for travel, lower exposure to communicable diseases, and help prevent deferred care (8, 9, 35). Telehealth further enables easier and faster access to providers, reduces appointment cancellations and no-shows, shortens wait times, and increases the opportunity for follow-up or in-depth conversations (8, 9, 35). Telehealth helps decrease emergency department utilization, hospitalizations, and healthcare costs, while improving efficiency for clinicians and facilitating outreach and education (8, 9, 35).

Quality of life for older adults is enhanced through telehealth supporting individuals' ability to age in place, defined as living safely, independently, and comfortably in one's own home and community, regardless of age, income, or functional ability (53). Aging in place is associated with reduced costs compared to institutional care, higher life satisfaction, improved health outcomes, and greater self-esteem (53). Receiving medical care at home promotes autonomy and independent living by offering a sense of safety and reassurance (36). Remaining at home correlates with increased dignity, independence, and quality of life, aligning with the growing preference among older adults to avoid facility-based aging (8, 9, 36). eHealth services further enhance well-being by fostering security through continuous monitoring, allowing older individuals to maintain familiarity and control in their living environment (36). Frequent use of telecare, alongside strong social and health support systems, is linked to higher quality of life and greater technology acceptance in this population (36).

Telehealth offers benefits for caregivers as well, including increased access to health technologies and decreased patient-caregiver stress (8, 9, 34). Caregivers found tele-dementia care convenient, comfortable, stress reducing, timesaving, and highly satisfactory. Caregivers prefer a combination of in-person and telehealth visits (41). Communication between providers and caregivers was enhanced through telehealth (35). Further, increased caregiver engagement through telehealth is noted (42). In contrast to in-person visits, where physical space limitations often make it difficult to separate the caregiver and person with dementia, telehealth facilitates caregiver education and care planning discussion without requiring the person with dementia to participate in the conversation (42).

**Figure 2:**
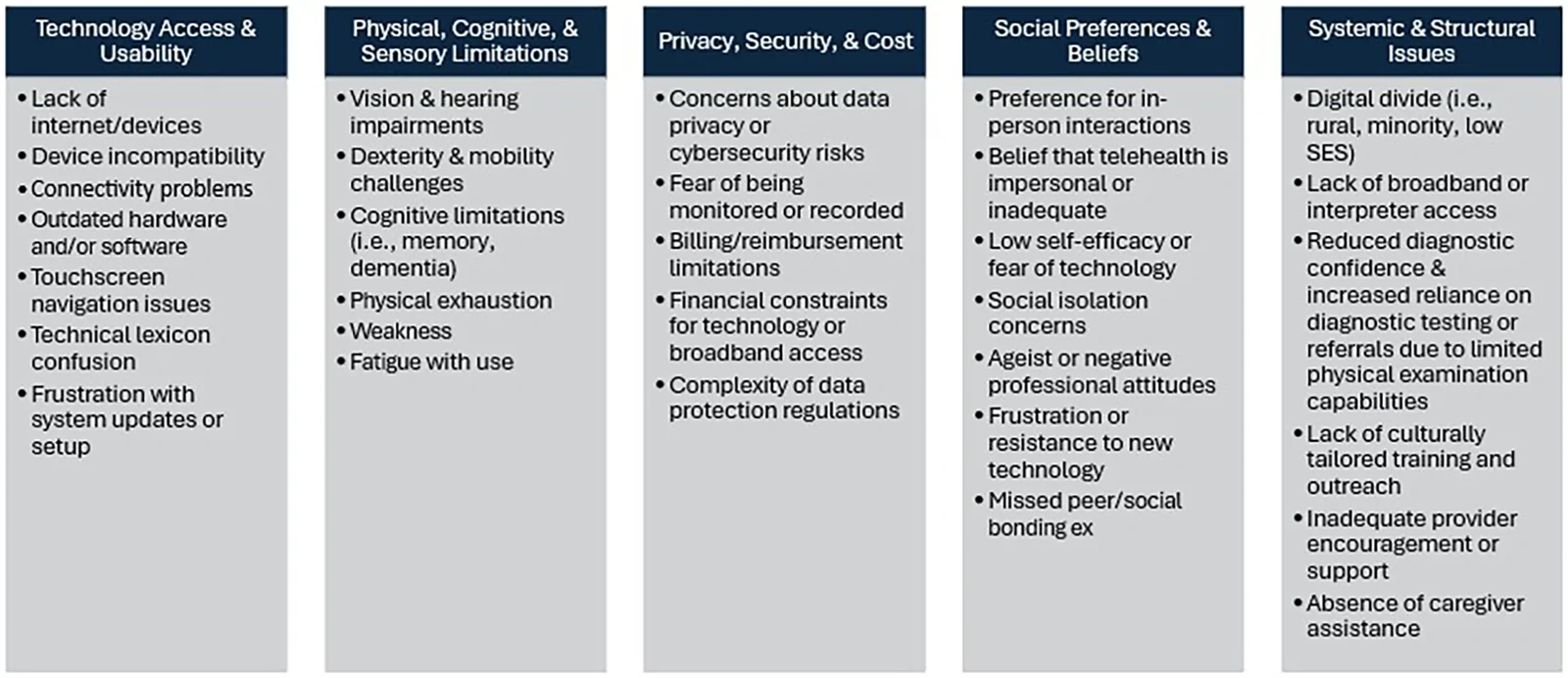
Barriers to telehealth adoption.

Despite the benefits, several challenges to telehealth adoption are noted in current peer-reviewed literature (8, 9, 13, 23, 26–28, 34–38, 40, 42, 46, 47, 50, 51, 53, 56, 58, 59, 62). Figure 2 provides a categorized summary of common challenges to telehealth use among older adults. These categories include: (1) Technology Access & Usability; (2) Physical, Cognitive, & Sensory Limitations; (3) Privacy, Security, & Cost Concerns; (4) Social Preferences & Beliefs; and (5) Systemic & Structural Issues. Virtual exercise interventions present barriers related to the home environment, privacy concerns, technological disruptions, difficulty assessing form through two-dimensional video, decreased engagement, and fewer opportunities for social interaction (40, 50). Despite these barriers, virtual interventions have been shown to effectively promote physical activity among older adults (55, 63). Evidence-based strategies to overcome these challenges include the use of adjustable device stands, HDMI connectivity to larger screens, sufficient verbal cues, closed captions, screen reader alerts, readable font sizes, and adjustable audio (50). Instructors should receive telehealth-specific training, and programs should consider pre-class usability testing, environmental assessments, and provision of technical support materials. Additional staff can serve as tech moderators, and structured opportunities for socialization before and after classes can help build community (40, 50). Implementing these strategies supports safer, more inclusive, and engaging telehealth-based physical activity programs for older adults.

Clinicians' attitudes significantly influence the adoption of telehealth among older adults. When clinicians perceive older adults as capable and willing to use telehealth, they are more likely to offer such services (8, 9). Some physicians view digital literacy as a diminishing barrier, expecting that as younger cohorts age, telehealth will become standard (35). Clinicians also emphasized that the benefits of access provided by telehealth often outweigh potential drawbacks (35). However, concerns remain. Some older physicians reported missing face-to-face interactions with patients and colleagues, which negatively impacted their morale (35). Others expressed skepticism about the ability of individuals with cognitive impairments, such as dementia, to effectively use video-based telehealth independently (34).

Older adults face significant and multifaceted barriers when accessing telehealth, ranging from limited technological access to complex system-level challenges. These barriers have persisted even as virtual care continues to expand, underscoring the need for intentional, evidence-based strategies to close the digital divide and ensure equitable care (35, 70). Technological challenges remain among the most prominent barriers. Many older adults lack access to smartphones, tablets, or computers, and even when these devices are available, they may not have reliable broadband connections or the digital literacy necessary to use them (33, 38). Navigating telehealth platforms, downloading apps, or troubleshooting software issues can be especially difficult for those unfamiliar with the technology. Furthermore, cognitive limitations, vision or hearing impairments, and physical dexterity issues can further complicate the telehealth experience, making it less accessible to those with age-related health conditions (33, 36).

Compounding these individual-level barriers are systemic social inequities. Racial and ethnic minorities, individuals with low income, and residents in rural communities disproportionately face obstacles to digital health access (25, 70). These groups often lack both the infrastructure and tailored support necessary for successful telehealth engagement. Health systems themselves may inadvertently contribute to access issues by underinvesting in technical assistance, failing to recognize patients' needs early in the care process, or applying rigid policies that exclude caregivers or informal support networks from telehealth participation (42, 48).

Research has highlighted numerous evidence-based strategies that can meaningfully improve telehealth access and engagement among older adults (Table 3). For example, providing tip sheets to clinicians, including guidance on distinguishing their role from that of a tech support figure, communication tips, and steps to help patients download and navigate apps—can empower providers to better support their patients (70). Proactive telephone outreach to patients at risk of deferring care has also proven effective in maintaining clinical contact (70).

**Table 3:** Barriers to telehealth use among older adults and evidence-informed mitigation strategies.

Barrier category	Examples	Evidence-Informed Strategies
1. Technology access and usability	Lack of internet, outdated devices, difficulty navigating platforms	Provide loaner tablets with preloaded software Use phone-based options for low-bandwidth settings Offer pre-session tech support and caregiver assistance
2. Physical, cognitive, and sensory limitations	Low vision, hearing loss, memory challenges, reduced mobility	Incorporate closed captioning and adjustable volume controls Use simple interfaces with visual cues and repetition Encourage caregiver involvement for safety and assistance
3. Privacy, security, and cost	Fear of surveillance, identity theft, concerns over cost of data or services	Use HIPAA-compliant platforms with transparent data policies Educate patients about privacy safeguards and control over camera/mic Partner with community orgs for financial assistance (e.g., low-cost internet)
4. Social preferences and beliefs	Preference for in-person interaction, skepticism about virtual care, mistrust of technology	Build rapport via consistent providers and small-group sessions Normalize virtual care through peer stories/testimonials Blend virtual and occasional in-person options to ease transition
5. Systemic and structural issues	Language barriers, digital redlining, lack of culturally tailored materials	Provide language translation and bilingual staff when possible Address internet inequity via mobile hotspots or device grants Tailor program content to cultural and community contexts

Health systems are encouraged to build partnerships with community organizations and government agencies to advocate for equitable access to devices, internet services, and digital literacy education (70). Supporting staff time for hands-on technology assistance, whether through scheduled training or on-demand help during telehealth visits, has been shown to significantly improve participation (13, 70). These efforts should be accompanied by policy changes such as payment parity for in-person and virtual visits, Medicare coverage expansion for remote services, and deregulation of restrictive licensing and HIPAA compliance requirements to support wider telehealth adoption (35). Addressing the medicolegal uncertainties associated with virtual care—such as fears about diagnostic errors—can also help clinicians feel more confident in embracing telehealth (35).

Patient-centered strategies are equally important. At the time of scheduling, staff should assess a patient's readiness and technological capacity to engage in virtual care (48). If gaps are identified, tailored interventions can follow, such as test calls, practice sessions, or distribution of user-friendly guides (38). Providing older adults with the opportunity to practice using telehealth platforms before a clinical visit helps build confidence, while real-time reminder calls ensure they log on and stay engaged (48). Programs that offer coaching and peer support are particularly beneficial for older adults who need repeated, personalized training (21, 34). The inclusion of care partners in telehealth planning provides an essential layer of help for those managing complex health or technological issues (38).

Several studies also emphasize the importance of designing telehealth systems and materials that consider the preferences and changing needs of older adults (36, 62). Training materials should be simple, accessible, and designed specifically for older users. Human support, in the form of hotlines, live technical assistance, or community-based educators, can often make the difference between a successful visit and a missed opportunity (13). Offering assistive technologies such as stylus pens, screen magnifiers, or built-in accessibility features can further reduce barriers. HDMI adapters may enhance accessibility by enabling digital content to be viewed on larger screens (29, 30). In-home technical assistance and easy-to-use video applications have emerged as highly effective interventions, especially for those with visual, cognitive, or functional impairments (33).

Ultimately, improving telehealth access for older adults requires more than just identifying those without devices or internet. It demands a structural and sustained commitment to reducing digital health disparities (26, 51). Health systems must continue to monitor telehealth use across populations to identify patterns of exclusion and develop responsive strategies (25). Clinicians, too, must be prepared to assist when technology falters or to refer patients to specialized support services when needed (61). When older adults are supported with the right tools, training, and policy frameworks, telehealth becomes not a barrier but a bridge—connecting them to essential care while honoring their unique needs, preferences, and dignity.

## 4 Discussion

### 4.1 Principal findings

This scoping review revealed a rapidly growing body of literature focused on older adults and the use of health technologies, particularly in the context of synchronous telehealth. Wardlow et al. (8, 9) underscores how the COVID-19 pandemic catalyzed this expansion. Consistent with this trend, most studies in this review were published between 2021 and 2024, reflecting the acceleration of digital health adoption during and following the pandemic. Most studies focused on the individual level, with more recent literature expanding to include team, facility, and system-level analyses. Despite this growth, there remains considerable variability in how older adults are defined, the populations studied, the types of technologies used, and the conceptual frameworks applied. This inconsistency limits the generalizability of findings and highlights the need for standardized definitions and methodology in future research.

The findings underscore the continued presence of the digital divide among older adults, shaped not only by age but also by intersecting factors such as cognitive ability, functional status, rurality, socioeconomic status, and veteran status. The findings also reveal gaps in addressing multilevel implementation challenges. The prevalence of studies involving veterans and individuals with chronic conditions or cognitive impairments illustrates the complex interplay between age, health status, and digital readiness. The limited use of theoretical frameworks and standardized assessments in a significant portion of the studies further points to a gap in rigorous, theory-informed evaluations of digital health interventions. Importantly, the emergence of studies addressing care at the system level indicates a growing recognition of the structural and policy-related barriers to digital health equity.

Although telehealth has become a critical component of healthcare delivery, multiple barriers continue to limit its equitable utilization. These barriers can be broadly categorized into patient-related, provider-related, technological, operational, and systemic factors. Patient-related challenges play a significant role in limiting telehealth engagement. Patients may be less likely to disclose important health information during telehealth visits compared to in-person consultations (15). Among older adults, physical limitations, sensory impairments (e.g., vision and hearing loss), cognitive decline, and low technology literacy present considerable obstacles (8–10, 64). Language barriers further exacerbate access difficulties, particularly when the coordination of interpretive services during telehealth encounters is more complex than during in-person care (10). Mental health conditions, such as substance use disorders and psychosis, may impair patients' abilities to engage meaningfully in virtual consultations (10). Patients’ perceptions also influence utilization; concerns that telehealth visits are shorter, less comprehensive, and less conducive to sharing broader health concerns are common (15). Additional deterrents include privacy and security concerns, distrust in healthcare providers or systems, and a general preference for in-person interactions (10, 64).

Despite advancements in telehealth, some clinicians continue to perceive a disparity in the quality and acceptability of virtual vs. in-person clinical encounters (10). This perception may stem from concerns regarding the limitations of virtual platforms in replicating the nuanced elements of face-to-face interactions, such as physical examinations and detection of non-verbal cues (i.e., body language, personal hygiene, presence of alcohol on the breath), and may limit discussions of multiple or complex health issues (10, 65). There is growing concern regarding the challenges associated with establishing rapport and cultivating strong clinical relationships in virtual settings, which may diminish the overall efficacy of care delivery (65). Certain providers face challenges in modifying treatment plans that rely on hands-on interventions and/or manual assessments, which necessitate direct patient interaction and cannot be effectively conducted via telehealth platforms (10). Moreover, safety concerns, particularly during tele- exercise visits, represent a significant barrier to telehealth utilization (66). The inability to provide immediate intervention in the event of a physical or mental health emergency raises critical challenges (10, 66). Some providers perceived an increased burden related to assisting with technology transition and associated care (10).

Reliable access to technology remains uneven, particularly among the economically disadvantaged or those living in rural areas. Barriers include limited access to affordable devices, unreliable internet connectivity, low technology literacy, and difficulties navigating telehealth applications (8–10, 64, 66). Technical failures during virtual encounters, such as poor video quality or dropped connections, can further discourage both providers and patients from utilizing telehealth services (10).

Telehealth implementation often introduces additional operational demands compared to traditional in-person care. Preparing physical spaces, coordinating the availability of appropriate technology, managing equipment across different locations, and allocating additional time for visit planning and troubleshooting introduce complexities not typically present during in-person encounters (65). These increased workflow demands can strain clinical operations and reduce the efficiency and scalability of telehealth services.

Systemic issues, including ambiguous billing guidelines, inconsistent reimbursement policies, and lower payment rates for telehealth services relative to in-person care, hinder the widespread adoption of virtual care models (10). Limited patient education and awareness regarding telehealth options also contribute to disparities in access (10, 64). In some geographic regions, particularly rural and underserved areas, the absence of local providers offering telehealth services further exacerbates the digital divide (10). Understanding the multifaceted barriers to telehealth utilization is essential for informing effective strategies aimed at improving access. Addressing these barriers requires coordinated efforts across patient education, provider support, technological development, operational processes, and systemic healthcare reforms.

### 4.2 Implications for practice and policy

The results of this scoping review point to several actionable implications for clinical practice, education, technological, and system-level policy. Although this scoping review highlights the limited application of theoretical frameworks in studies on digital health for older adults, several notable exceptions illustrate person-centered, age-inclusive models. Wardlow et al. (8, 9) reviewed telehealth principles and guidelines specifically tailored for older adults, emphasizing person-centered care, equitable access, and integrated service delivery. These guidelines address physical, cognitive, cultural, and technological barriers, promote caregiver involvement, and support continuity of care, offering a comprehensive framework for the development and evaluation of digital health interventions for aging populations. Additionally, the 4Ms Framework (What Matters, Medication, Mentation, and Mobility) emerged as a relevant model, underscoring the impact of the digital divide on the delivery of age-friendly care (8, 9, 39, 49, 60). While healthcare teams should align closely with the four evidence-based elements of high-quality care for older adults in healthcare systems, such as the 4Ms Framework, in the current scoping review only four (8.9% of studies) included any mention of this framework. Disparities in digital access and literacy may undermine the effectiveness of technology in supporting personalized care goals, medication management, cognitive health, and mobility promotion. The integration of theoretical frameworks into research, clinical practice, and operational strategies that support patients during all stages of an encounter (pre-visit, intra-visit, and post-visit) is essential for advancing equitable and effective digital health solutions for older adults (71).

To support the successful implementation of these frameworks, training initiatives for both consumers and providers are essential as they bridge knowledge gaps, reduce inequities, and support the capacity of healthcare systems to meet the needs of all population groups, including those at risk of being underserved (10, 67). It is equally critical that clinical teams receive ongoing, structured training that prepares them to address the wide range of cognitive, functional, sensory, and technological needs common among older adults. This includes not only understanding the physical and cognitive limitations that may affect technology use but also possessing the skills to assess digital literacy and to deliver patient and caregiver centered education tailored to individual abilities and preferences. Training should emphasize communication strategies that promote patient engagement, foster caregiver involvement, and accommodate sensory or cognitive impairments. Furthermore, education programs should equip clinicians to identify and mitigate structural barriers such as lack of broadband access or device availability and to advocate for system-level policies that promote digital inclusion. Without this comprehensive, multidisciplinary approach to workforce preparation, digital health interventions risk exacerbating, rather than narrowing, disparities in care for older populations.

Facilitators of telehealth adoption and effectiveness for older adults and other patient populations include a variety of technology solutions and supportive practices. Simple user interfaces, large fonts, adjustable audio capabilities, and voice-to-text or text-to-voice features help make devices more accessible for those with limited tech experience or physical impairments (8, 9). Additionally, the use of simpler technologies like audio-only visits, combined with tailored training programs, promotes engagement and reduces barriers to entry (8, 9). Hotspots, caregivers, and telehealth facilitators who assist with setup at patients' homes further enhance access to care, including through straightforward solutions like using a telephone or asking family members for help (8, 9). In the context of mental health and physical health, practices such as orienting patients to telehealth platforms, offering flexible staff support, and integrating in-person and virtual visits through electronic health records (EHRs) help improve the overall telehealth experience (65). Facilitators for mobile app utilization include patients’ positive perceptions of app usefulness, encouragement from trusted sources, and the app's ability to provide data visualizations or track health goals (64). Additionally, pre-established relationships with healthcare providers, tracking patient demographics, and incorporating patient satisfaction surveys contribute to improved engagement and identification of underserved groups (15).

The findings of this scoping review also underscore the need for implementation strategies that extend beyond the individual to consider contextual, structural, and policy-level influences. Bridging the digital divide for older adults will require intentional design, tailored support, and multi-level interventions that align with the lived realities and need of aging populations. At the system level, there is a need for infrastructure investments (e.g., free or low-cost access to telecommunications technologies) that ensures equitable access to broadband internet and devices, particularly in underserved or rural communities (67). Policy makers should prioritize reimbursement models and regulatory frameworks that support integrated, age-friendly digital care across settings. Advocacy should focus on marginalized and diverse populations, while research guides telehealth funding and adoption (67). Equitable telehealth requires culturally competent policies that support change and innovation in care delivery (67). Programs including translation services and those designed to deliver care to older adults in diverse living situations (i.e., traditional homes, congregate housing, long-term care facilities) increase access to telehealth services (8, 9, 67).

Efforts to mitigate barriers to telehealth utilization among older adults and digitally marginalized populations have centered on several interrelated strategies. A primary approach is increasing consumer knowledge of telehealth services by developing targeted marketing and communication materials, available in multiple languages, to enhance awareness and understanding (67). Early and continuous consumer engagement in the design of telehealth services is critical and includes co-designing culturally competent services, integrating consumer-reported experience measures, and offering incentives to encourage collaboration in the development of care models that align with consumer needs and expectations (67).

Improving access to technological devices remains a priority (10, 14). Strategies include developing device loan programs, providing culturally appropriate information tailored to daily living practices, and introducing interactive technologies to foster active engagement and self-management skills (67). Practical support mechanisms, such as offering digital literacy training, providing technical assistance for both patients and providers, and facilitating shared decision-making regarding telehealth use, further empower patients (10). Facilitating patient referrals to community resources that support technology access and telehealth literacy, alongside establishing community partnerships to develop internet hubs, can collectively enhance telehealth utilization, particularly among populations at risk for digital exclusion (64, 68). Adopting hybrid models of care, which integrate both telehealth and in-person visits, has the potential to significantly increase telehealth utilization (10, 14). However, to ensure the feasibility of such models, particularly for Medicare beneficiaries who may face transportation barriers, it is imperative to strengthen Medicare-funded transportation services (14).

High-level strategies to improve telehealth access and utilization include prioritizing research focused on under-resourced populations (i.e., culturally and linguistically diverse groups, individuals with low socioeconomic status, and those with limited health literacy), expanding coverage for telehealth services, and increasing reimbursement for telehealth care delivery (10, 67). Other high-level strategies, including expanding broadband access, increasing reimbursement for telehealth services, broadening cross-state licensure for health professionals, and improving interpreter services integration are important systemic measures to reduce barriers (10).

Strong patient-provider relationships, simplified and intuitive technology designs, clear instruction manuals, and the use of familiar communication platforms enhance the usability and acceptability of telehealth services among older adults (14). Traditional outreach methods (e.g., mailed letters, posters) and integrating telehealth into routine care processes (such as using patient portals to drive adoption of health-related apps) serve as low-barrier interventions to improve telehealth adoption across diverse populations (14, 64).

### 4.3 Limitations

This review has several limitations. Consistent with scoping review methodology, a formal quality appraisal of included studies was not conducted. Consequently, the methodological rigor, risk of bias, and overall strength of the evidence were not systematically evaluated. The findings should be interpreted as a synthesis of the existing literature rather than an evaluation of reported interventions and outcomes.

A geographic limitation of this review is the concentration of included studies in the United States, which accounted for 84.4% (*n* = 38) of the final sample. The remaining studies represented seven other countries and one global/multinational study. This geographic skew substantially limits the generalizability of findings to international contexts. Digital infrastructure, healthcare system design, reimbursement models, broadband access, and aging demographics vary considerably across countries, and strategies that are feasible or effective within the United States healthcare system may not translate directly to other national contexts. Findings related to policy, reimbursement, and systemic implementation may reflect U.S.-specific conditions that do not apply broadly.

An additional challenge was the lack of consistency in how “older adults” were defined across studies. Age thresholds varied considerably, which may have influenced reported experiences, levels of digital literacy, technology adoption, and engagement with digital health interventions. This heterogeneity complicates comparisons across studies and limits the ability to synthesize findings into a cohesive understanding of age-related digital health disparities. The limited use of validated theoretical frameworks and standardized assessment tools across a significant portion of included studies further constrains the ability to draw definitive conclusions regarding age-related disparities in digital health technology use.

### 4.4 Future directions

Future research should prioritize addressing the methodological and structural gaps identified in this review. Standardized definitions of “older adult,” consistent theoretical frameworks, and validated assessment tools are needed to improve comparability across studies and strengthen the evidence base for digital health interventions. Future studies should adopt a clear rationale for age-based inclusion criteria and report findings by age subgroups, when appropriate, to support the development of targeted interventions and policies that address the needs of diverse older adult populations. Additionally, systematic reviews that include formal quality appraisal are needed to evaluate the strength of the evidence and support more definitive conclusions.

Longitudinal research examining the sustained impact of telehealth adoption among older adults, particularly those with cognitive impairments, sensory limitations, or low digital literacy, would meaningfully advance the field. Greater attention should be directed toward under-resourced and historically marginalized populations, including rural communities, veterans, and culturally and linguistically diverse groups, to ensure that future interventions are equitable and inclusive. Future research should also intentionally include diverse geographic settings, with particular attention to low- and middle-income countries where aging populations are growing rapidly and digital health inequities may be most pronounced.

At the systems level, research is needed to evaluate the effectiveness of policy reforms such as expanded broadband access, cross-state licensure, and reimbursement models in reducing the digital divide. Future studies should employ multilevel designs that capture individual, team, facility, and system-level outcomes simultaneously, reflecting the complex and interconnected nature of digital health implementation in aging populations. Addressing these areas in practice, policy, and research will support more inclusive, effective, and equitable digital health care for older adults.

## 5 Conclusions

This scoping review highlights both the promise and the persistent challenges of digital health technology adoption among older adults. While telehealth has emerged as a vital modality for healthcare delivery, particularly in the wake of the COVID-19 pandemic, its equitable utilization remains constrained by a complex web of individual, provider, technological, operational, and systemic barriers. Addressing these barriers demands a coordinated, multilevel response that integrates person-centered frameworks, targeted workforce training, infrastructure investment, and inclusive policy reform. The variability identified across studies in how older adults are defined, the populations examined, and the frameworks applied underscores the urgent need for standardization in both research methodology and clinical implementation. Moving forward, the field must prioritize the voices and lived experiences of underserved older adult populations, including those with cognitive impairments, limited digital literacy, and rurality, to ensure that advances in digital health translate into meaningful, equitable improvements in care. By aligning research rigor with actionable policy and practice, the healthcare community can work toward a future in which age, geography, and socioeconomic status are no longer determinants of one's ability to access and benefit from digital health services.
